# Drug drug interaction extraction from the literature using a recursive neural network

**DOI:** 10.1371/journal.pone.0190926

**Published:** 2018-01-26

**Authors:** Sangrak Lim, Kyubum Lee, Jaewoo Kang

**Affiliations:** 1 Department of Computer Science and Engineering, Korea University, Seoul, Korea; 2 Interdisciplinary Graduate Program in Bioinformatics, Korea University, Seoul, Korea; National Chiao Tung University College of Biological Science and Technology, TAIWAN

## Abstract

Detecting drug-drug interactions (DDI) is important because information on DDIs can help prevent adverse effects from drug combinations. Since there are many new DDI-related papers published in the biomedical domain, manually extracting DDI information from the literature is a laborious task. However, text mining can be used to find DDIs in the biomedical literature. Among the recently developed neural networks, we use a Recursive Neural Network to improve the performance of DDI extraction. Our recursive neural network model uses a position feature, a subtree containment feature, and an ensemble method to improve the performance of DDI extraction. Compared with the state-of-the-art models, the DDI detection and type classifiers of our model performed 4.4% and 2.8% better, respectively, on the DDIExtraction Challenge’13 test data. We also validated our model on the PK DDI corpus that consists of two types of DDIs data: *in vivo* DDI and *in vitro* DDI. Compared with the existing model, our detection classifier performed 2.3% and 6.7% better on *in vivo* and *in vitro* data respectively. The results of our validation demonstrate that our model can automatically extract DDIs better than existing models.

## Introduction

Drug-drug interactions (DDIs) may occur when two or more drugs are co-administered, and thus the effects of the combined drugs can be increased, weakened, or harmful. It is known that such DDI events may cause preventable drug related harm [1]. Several databases such as DrugBank [2], PharmGKB [3], Drugs.com [4] and Stockley’s Drug Interactions [5] collect known adverse events caused by DDIs. Usually, human experts manually collect DDI information from various sources such as the FDA’s Adverse Event Reporting System [6]. Since there are numerous combinations of drugs available, it is difficult to collect all the DDI events of patients from reports or publications. Also, manually organizing DDI information in natural language into a DDI database is costly and time-consuming.

Several efforts to automatically collect DDI information from the biomedical literature using text mining tools have been made. The DDI Challenges in 2011 and 2013 [7] released gold standard datasets for the task of improving the performance of DDI extraction using a Natural Language Processing (NLP) pipeline. Using support vector machines (SVMs), some of the methods obtained better results on these datasets [8, 9]. Unfortunately, the methods that use traditional machine learning classifiers such as SVMs require feature engineering of domain experts, which is also expensive and time consuming. However, several recent deep learning methods have achieved comparable results without using feature engineering [10–14].

In this paper, we build a DDI extraction model using a Recursive Neural Network based approach. Our recursive neural network model in NLP uses syntactical features of each node in a parse tree. Since the grammatical structure of natural language sentences is known to be recursive [15], we believe a recursive neural network model would be more effective for understanding the DDI-related sentences and extracting information from them.

Socher et al. [16] proposed a Matrix-Vector Recursive Neural Network (MV-RNN) model that assigns a vector and a matrix to every node in a parse tree to classify the relation of two target nouns in a sentence. They showed that their recursive neural network model is effective for finding relations between two entities. Unfortunately, the the MV-RNN model’s performance on the DDI extraction task was unsatisfactory [17]. However, in this paper, we show that our recursive neural network model can improve the performance of DDI extraction using additional features. The parse tree of a sentence alone is insufficient to convey the location of target drug pairs. We use a position feature, a subtree containment feature, and an ensemble method for improving the performance of DDI extraction in this study.

We validate our model on two different corpora: the DDI’13 corpus and the PK DDI corpus. DDI’13 corpus is the most widely known and manually annotated corpus among the DDI-related corpora. The PK DDI corpus is also manually annotated [18]. Both corpora aim to support the development of DDI extraction techniques using NLP. DDIs have roughly two types of interactions: pharmacokinetics (PK) and pharmacodynamics (PD). Pharmacokinetics is the study of what the body does to a drug including processes from drug absorption to excretion. On the other hand, pharmacodynamics focuses on the effects of drugs on organisms. The DDI’13 corpus contains both PK and PD types of interactions, and the PK DDI corpus contains only PK-type interactions.

## Materials and methods

### Model development

The SemEval 2013 task 9.2 has two objectives. The first focuses on detecting positive DDIs in all possible pairs of drugs and the second focuses on the multi-class type classifier of each positive DDI pair of one of the following four types: advice, effect, mechanism, and int. The DDI types are explained in Table 1. The one-stage method, one of the existing DDI extraction methods [19], performs detection and type classification at the same time by classifying the negative instance and the four DDI types at once. The two-stage method, another DDI extraction technique, divides the process into detection and type classification [8]. The first detection stage involves determining whether an interaction between two drugs exists. In the second type classification stage, the model receives the predicted positive pairs from the first stage as input and performs multi-class classification to determine the types of DDIs. The given data has more negative relations than positive relations, and each positive relation is one of the four relation types mentioned above. Since the detection classifier of the two-stage method does not divide the positive instances by type, the full set of positive instances can be utilized to train the detection classifier. We implement both the one-stage and the two-stage methods.

**Table 1:** DDI relation types and explanations.

Types	Explanation
Advice	This type is assigned when a sentence contains recommendation or advice regarding the concomitant use of two drugs
Effect	This type is assigned when a sentence contains pharmacodynamic mechanism including a clinical finding, signs or symptoms, an increased toxicity or therapeutic failure.
Mechanism	This type is assigned when a sentence contains pharmacokinetic mechanism including changes in levels or concentration of the entities.
Int	This type is assigned when a sentence states that an interaction occurs and does not provide any information about the interaction.

**Fig 1:**
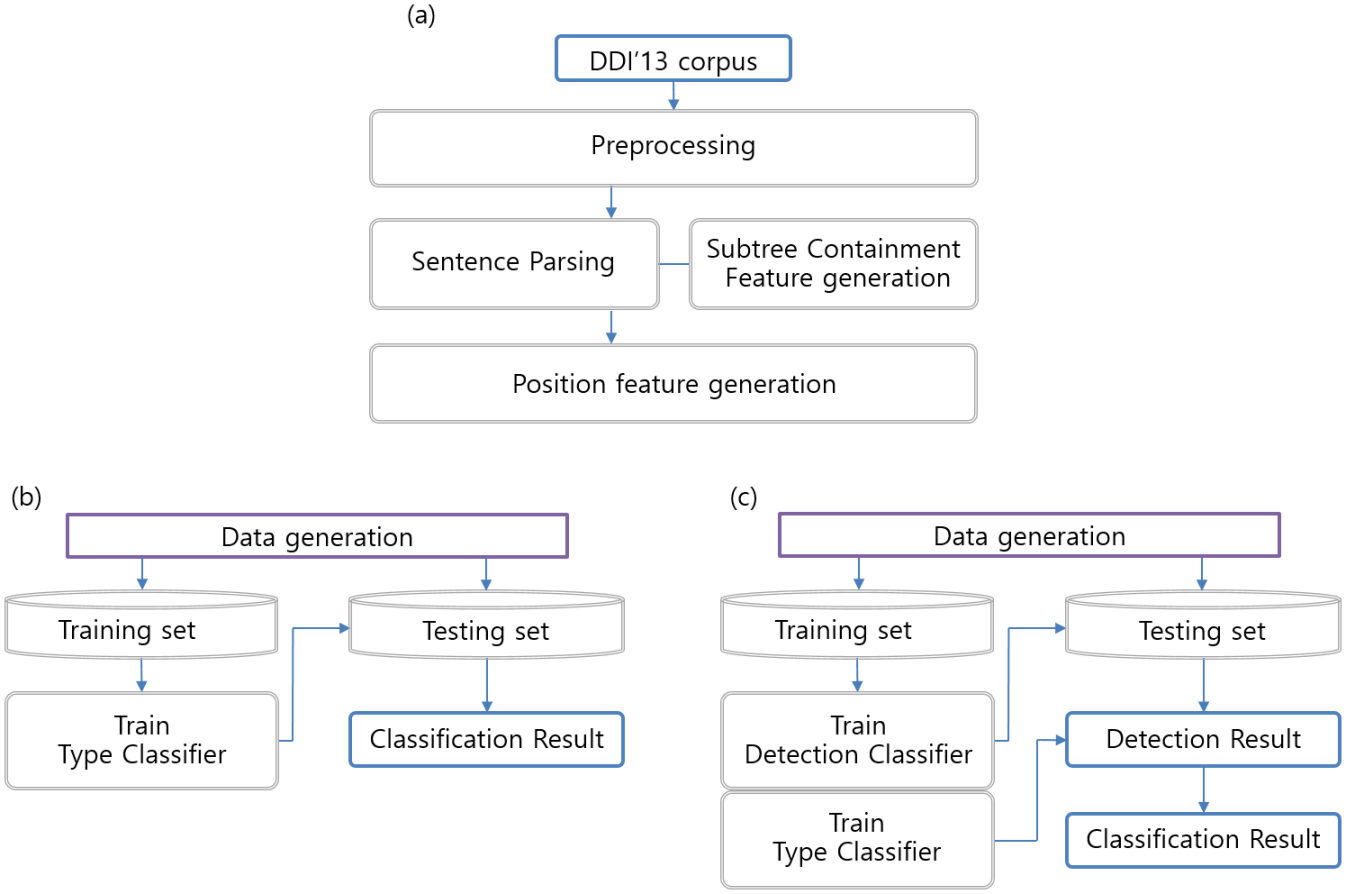
Overall system architecture. We implemented both the one-stage and the two-stage method. (a) Data generation part. (b) One-stage method. Five-class type classifier for the one-stage method. (c) Two-stage method. The DDI detection classifier distinguishes positive DDI instances from negative instances. The DDI type classifier receives the predicted positive instances from the detection classifier as a testing set.

Our overall system architecture is presented in the Fig 1. In the data generation part (Fig 1a), we apply the preprocessing first to improve the performance. Because our recursive neural network model takes parsed sentences as an input, we use the NLP library to parse the given sentences. During the parsing process, the subtree containment feature is generated. In the position feature generation step, we obtain the relative distance between each word and the target drugs. The target drugs are any pair of drugs in a sentence, and are the current focus of DDI extraction. The DDI detection task evaluates all possible pairs of drugs in a sentence to determine DDI relations. After the position feature generation step, we label the training data for both detection and type classification tasks. In the two-stage method (Fig 1c), our detection and type classifiers share the same model with different inputs and outputs. The detection classifier does not learn labels for type classification and vice versa. The detection testing should be performed prior to the type classification testing because only the predicted positive pairs in the DDI detection result are used as type classifier testing data. On the other hand, the one-stage method (Fig 1b) uses five-class type classifier without the detection stage. Since the PK DDI data does not have DDI-type information, we use only the detection classifier on the PK DDI data.

### Preprocessing

Preprocessing involves removing obvious noise, tokenization, and anonymizing target drugs. These steps reduce the size of the vocabulary and help boost the performance.

#### Tokenization, replacement and anonymization

In the task of extracting DDIs, most drug names in sentences are extracted and annotated as entities. However, not all entities in sentences are annotated. In the biomedical domain, an entity can have multiple names; therefore, we converted entity names to more commonly used names to effectively use word embedding. We employed the biomedical entity extractor used in Biomedical Entity Search Tool (BEST) (http://infos.korea.ac.kr/bioentityextractor/) to find and replace entity names with more commonly used terms. BEST aims to complement standard biomedical search tools such as PubMed [20].

Because the DDI’13 corpus is employed for extracting DDIs using NLP, a target drug pair is labeled as “False” if its interaction is not represented in a sentence, even though there is an actual interaction between the two drugs. Drug names do not play a significant role in the DDI detection process; therefore, earlier studies replaced the drug names with designated names such as “*Ddrug*0” for the first drug and “*Ddrug*1” for the second drug and so on [8–11]. We followed the same strategy for our study. Replacing drug entities with designated names also addresses the unusual cases where target entities are composed of two or more non-sequential words. For instance, the first sentence in the following example, is changed to the second sentence. In the first sentence, both words “nonheme” and “heme” are connected with “iron” by a conjunction. The second target entity “nonheme iron” is changed to “Ddrug1” in the second sentence. The underlined words are the target drugs.

1. “Calcium_*drug*0_ is the only known component in the diet that may affect absorption of both nonheme_*drug*1_ and heme_*drug*2_ iron_*drug*1/2_.”
2. “Ddrug0 is the only known component in the diet that may affect absorption of both Ddrug1 and Ddrug2.”

After all the preprocessing steps, we changed all the independent numbers to “#” regardless of whether they were normal integers or floats.

#### Negative instance filtering

For a fair comparison, the data we use is almost the same as the data used in the previous study [10]. We did not perform the negative instance filtering ourselves, but we obtained the data IDs from the released code of the previous study and used the data with the same IDs. In this section, we briefly mention the filtering method applied to the data. In machine learning, imbalanced data degrades performance; thus, several studies that used the DDI’13 corpus implemented the following two rules to filter negative instances, and thus prevent performance degradation. The first rule is to remove any drug pair that refers to the same drug. This kind of drug pair may have the same drug name or synonyms. The second rule is to filter pairs of drugs that share coordinate relations. A coordinate relation refers to the case where two words are connected by a conjunction (e.g., “and,” “or”) a comma. Kim et al. [8] suggest that the pair of drugs in the same noun phrase have a coordinate relation. In many cases, the coordinate relation between three or more drugs is the feature of the negative instance.

### Parsing sentences

The constituency parse tree of a sentence contains syntactic interpretations of the sentence. For this reason, many existing papers have utilized results of constituency parsers. However, sequential models cannot use constituency parse trees to the full extent. The Long Short-Term Memory (LSTM) model constructs a hidden state from the input vector of the current time step and from the hidden state / memory cell of the previous time step [21]. On the other hand, the tree-LSTM model receives the hidden state / memory cell from multiple nodes (children nodes) at the same time. For example, consider a sentence with a coordinating conjunction that connects two clauses. The recursive neural network model can tell which phrases are equivalent in the hierarchical structure of the constituency parse tree of a sentence. We use the Stanford Parser [22] to transform a sentence into a constituency parse tree. After the parsing process, we use the “binarizer” provided by the Stanford Parser to convert the constituency parse tree into a binary tree.

We calculate the subtree containment feature in the parsing stage. Since the subtree containment feature is converted into a vector by our recursive neural network model, we briefly explain how the feature is calculated; however, the details are discussed in the sections below. When one of the target drugs exists in the leaves of the current node, the subtree containment feature is given a value of one (context:true); otherwise, it is given a value of zero (context:false).

### Word embedding

Word embedding is a set of low-dimensional continuous vectors that are trained by an unsupervised language model. A word from the vocabulary of interest is mapped to a vector of real numbers using word embedding. Word embedding combined with a neural network is a method widely used to improve NLP performance [23, 24]. The vector form of words expresses the relationship between the words and it is used to enhance the performance of an NLP task with specific purposes including sentiment classification and relation extraction. After our model receives input words, each input word is mapped to pre-trained word embedding vectors by the lookup process. If the word embedding set does not contain an input word, we generate a random vector for the input word.

We used the PubMed-and-PMC-w2v word embedding, which is obtained from published materials (http://evexdb.org/pmresources/vec-space-models/) [25]. The word embedding is initialized with Word2vec using gensim [26]. The total vocabulary size of the word embedding is 4,087,446, and we use the words in the DDI’13 corpus only. The dimension size of the word embedding is 200.

### Recursive neural network with treeLSTM

**Fig 2:**
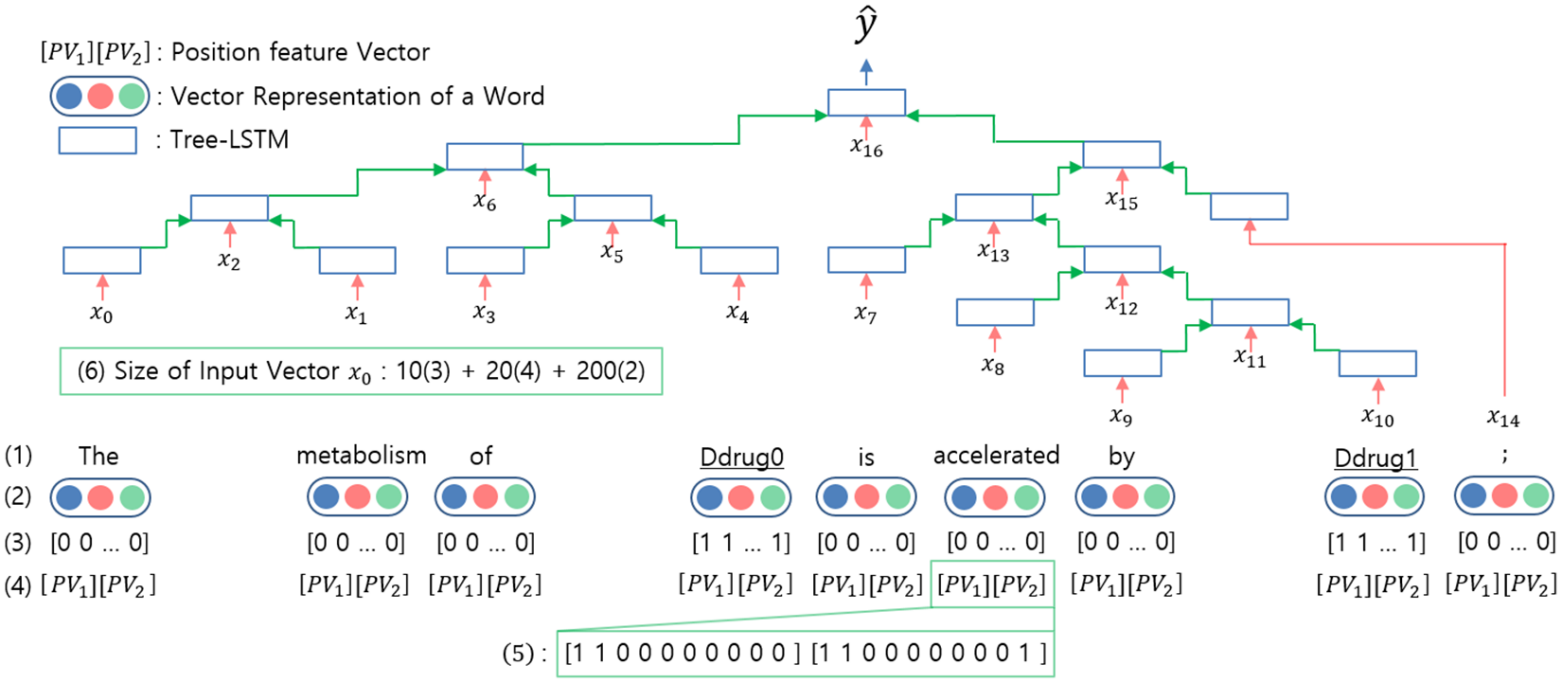
The architecture of our recursive neural network model. Our model is a variation of the binary tree-LSTM model. (1) The words in a sentence. The names of drug targets are underlined. (2) Vector representation of a word through the word embedding lookup process. (3) Subtree containment feature represents the importance of a node. (4) Position feature vector representing the relative distance of two target drugs from the current word position. (5) An example of the position feature vector. The current word is “accelerated.” (6) The size of the concatenated vector input *x*_0_ of our model is 10 (size of the subtree containment feature; (3) in the figure) + 20 (size of the position feature; (4) in the figure) + 200 (size of the word embedding; (2) in the figure).

The LSTM architecture [21] is a popular variation of the recurrent neural network that is capable of processing variable sized sequential data, such as sentences. To apply the LSTM architecture to the tree-structured information propagation, a tree-LSTM model was developed [27]. The tree-LSTM model can update the hidden state from the output states of more than one child node. In addition, there is a forget gate for every child node, so the model can select and integrate information from each child. The whole architecture of our model is presented in Fig 2.

After receiving a parsed input sentence (parse tree) to train our model, we look up the pre-trained word embedding to map each input word to real-valued vectors. If a node is not a leaf, the word representation vector is randomly initialized. Our model is based on the recursive neural network architecture of the child sum tree-LSTM model in [27, 28]. Let *x*_*j*_ denote the concatenation result of the vector representation of a word in a sentence with feature vectors. For any node j, we have two forget gates for each child and write the sub-node expression of the forget gates for k-th child as *f*_*jk*_. The *B*(*j*) is the set of values (including *h*_*k*_ and *c*_*k*_) from children of node j; since we use a binary tree, the size of *B*(*j*) is 2. *i*, *f*, *o*, *c*, *h* are the input gate, forget gate, output gate, memory cell, and the hidden state, respectively. *u* is a temporary value that could be added to the memory cell state. *drop*(*x*) in Eq (8) is a recurrent dropout [29] function. The *mask* is a sampled vector from the random distribution with probability *keep*_*p* that is used to decide which element is kept or dropped. The binary tree-LSTM equations are described below.

$$\tilde{h_{j}}=\sum_{k\in B(j)}h_{k},$$ (1)

$$i_{j}=\sigma (W^{i}\left[x_{j},\tilde{h_{j}}\right]+b^{i}),$$ (2)

$$f_{jk}=\sigma (W^{f}\left[x_{j},h_{k}\right]+b^{f}),$$ (3)

$$o_{j}=\sigma (W^{o}\left[x_{j},\tilde{h_{j}}\right]+b^{o}),$$ (4)

$$u_{j}=tanh(W^{u}\left[x_{j},\tilde{h_{j}}\right]+b^{u}),$$ (5)

$$c_{j}=i_{j}⊙drop\left(u_{j}\right)+\sum_{k\in B(j)}f_{jk}⊙c_{k},$$ (6)

$$h_{j}=o_{j}⊙tanh\left(c_{j}\right)$$ (7)

$$\begin{array}{cc} drop(x) & =\{\begin{array}{cc} mask*x,\;\; & if\;train\;phase,\\ x, & otherwise \end{array} \end{array}$$ (8)

We use a fully-connected layer as the output layer in Eqs (9) and (10). The fully-connected layer output size is the number of classes (2 for detection, 5 for type classifier). At each node j, we choose the predicted label $\hat{y_{j}}$ for a given output. However, since the predicted value of the internal nodes in the tree is not important, we take only the predicted values extracted from the root node of the entire sentence when the final score is calculated.

We use the softmax cross-entropy classifier to calculate the cost function in Eq (11). *m* is the total number of items in the training set. To alleviate the class imbalance problem, we modified the loss function of the detection classifier so that the positive instances were given a three times greater loss than the negative instances.

$$\begin{array}{cc} \hat{p}\left(y|x_{j}\right) & =W^{\left(fc\right)}h_{j}+b^{\left(fc\right)} \end{array}$$ (9)

$$\begin{array}{cc} \hat{y} & =\text{arg}\;\max_{y}\hat{p}\left(y|x_{j}\right) \end{array}$$ (10)

$$J(\theta )=-\frac{1}{m}\sum_{k}^{m}y^{k}log\left(softmax\left(\hat{p}\left(y^{k}|x^{k}\right)\right)\right)$$ (11)

We use the Adam optimizer [30] for gradient descent optimization. In the next sections, we present two features that we used to improve the performance of our model.

#### Position embedding feature

It is helpful to identify which words are the two target nouns of interest. Several existing studies [10, 31] used position embedding to represent the relative distance of two target drugs from each word position in a sentence.

Every word in a sentence has two relative distances, [*d*_1_, *d*_2_], where *d*_*i*_ is the relative distance to *i*_*th*_ target drug from the current word. For instance, in the sentence shown in Fig 2-(1), *d*_1_ is -2 and *d*_2_ is 2 as the current word (“accelerated”) is located two words behind the first drug name and two words before the second drug name. In the training phase, each relative distance is converted into a vector with a size of 10 according to the relative distance to a target drug. Since there are two distances, the total vector size of the position feature is 20. Table 2 shows the vector representation based on the relative distances.

**Table 2:** Vector representation according to the distance between one of the target drugs and a current word.

relative distance	-5	-4	-3	-2	-1	0	1	2	3	4	5	6–10	11–15	16–20	21–∞
	0	0	0	0	0	0	1	1	1	1	1	1	1	1	1
	0	0	0	0	0	0	0	0	0	0	0	0	0	0	1
	0	0	0	0	0	0	0	0	0	0	0	0	0	1	1
	0	0	0	0	0	0	0	0	0	0	0	0	1	1	1
	0	0	0	0	0	0	0	0	0	0	0	1	1	1	1
	1	0	0	0	0	0	0	0	0	0	1	1	1	1	1
	1	1	0	0	0	0	0	0	0	1	1	1	1	1	1
	1	1	1	0	0	0	0	0	1	1	1	1	1	1	1
	1	1	1	1	0	0	0	1	1	1	1	1	1	1	1
	1	1	1	1	1	0	1	1	1	1	1	1	1	1	1

Note: When the distance difference is 5 or less, the vector is assigned to each difference value. If the distance is greater than 5, the same vector is given in units of 5. We skip the columns ranged from -6 to -∞ of the relative distance due to space limitation.

Since our recursive neural network model processes parse trees rather than sentences, after the input sentence is parsed into a tree, the leaf nodes have position features, but the internal nodes lack the relative distance information. We address the problem by choosing the smaller absolute value of the relative distance of children nodes.

#### Subtree containment feature

We designed the subtree containment (context) feature to represent the importance of nodes. Context words around target drugs are important features in the sequential models [8, 10]. We changed the context concept to be compatible with the recursive neural network architecture. When one of the target drugs exists in the leaves of the current node, this feature is given a value of one (context:true); otherwise, it is given zero (context:false). In the training phase, this feature is transformed into a vector of size 10. If the value is one, every element in a vector is one; otherwise, every element in a vector is zero, such as the case in Fig 2-(3).

An input vector of a node in a tree uses the subtree containment feature vector, the position feature vector, and the vector representation of a word in a given sentence. The size of the whole input vector *x*_*j*_ is 10 + 20 + 200.

#### Regularization

The original tree-LSTM model [27] used *l*_2_ regularization. Later, the tree-LSTM model was implemented with the TensorFlow fold library [28] using recurrent dropout [29] instead of the *l*_2_ regularization. Recurrent dropout is a dropout variant that improves performance by minimizing memory loss which is especially more common when dropout is applied to a recurrent neural network. We used recurrent dropout instead of *l*_2_ regularization, and found recurrent dropout to be effective.

## Results

### Experimental settings

We use TensorFlow [32] to implement our models. TensorFlow version 1.1 is a popular open source library for machine learning and deep learning. The code is written in Python 3.4. We implement our model using TensorFlow Fold located at the following link (https://github.com/tensorflow/fold). Most deep learning libraries such as TensorFlow assume machine learning models are static, which makes it difficult to use them with dynamic structured models (e.g., recursive neural network). The TensorFlow Fold is specifically designed to deal with this problem.

#### Hyperparameter

As the DDI datasets do not have a standard development set, we conducted five-fold cross validation using the training set to select the optimal parameters. Table 3 illustrates the hyperparameter search process. We found the optimal parameters by moving one parameter within the specified test range by a specified test unit while other parameters were fixed. The epochs are the stopping point for each task. It is interesting to note that the epoch was set to 30 in the PKDDI *in vitro* dataset because of the large variation of validation performance. Zhang et al. [33] pointed out that the *in vitro* dataset has more complex sentences than the *in vivo* dataset.

**Table 3:** Search process to find the best hyperparameters used for our model.

	Parameter	Test Range	Test Unit	Selected
Common	Hidden Unit Size	64–256	32	128
	Subtree Containment Size	1–10	1	10
	Batch Size	100–200	20	100
Binary tree-LSTM	Learning Rate	0.0005–0.005	0.0001	0.0008
DDI’13 Detection	Keep Probability	0.5–1.0	0.05	0.75
	Detection Epoch	30–150	10	100
Binary tree-LSTM	Learning Rate	0.0005–0.005	0.0005	0.0007
DDI’13 Classification	Keep Probability	0.5–1.0	0.05	0.9
	Classification Epoch	30–150	10	130
Binary tree-LSTM	Learning Rate	0.0005–0.005	0.0005	0.003
PK DDI in vivo	Keep Probability	0.5–1.0	0.05	0.75
	Classification Epoch	30–150	10	80
Binary tree-LSTM	Learning Rate	0.0005–0.005	0.0005	0.001
PK DDI in vitro	Keep Probability	0.5–1.0	0.05	0.6
	Classification Epoch	30–150	10	30

Note: We found the optimal parameters by moving one parameter within the specified range by a specified unit while the other parameters were fixed. The keep probability is used for the dropout. The epochs are the stopping points for each task.

### DDI’13 data

In the DDI’13 corpus, the number of negatives is six times larger than the number of positives. Imbalanced data is a major cause of poor performance in machine learning. Most of the high-performance studies used negative instance filtering to improve the performance of machine learning. However, after the preprocessing step, the final dataset sizes of each study were different. We used almost the same number of data pairs from the released code and data of the previous study [10]. Nevertheless, some inevitable differences exist because we removed duplicate sentences from the training and the test set. We did not change the training data; however, the size of the training data in the paper [10] is different from that of the released data. The positive drug pairs removed from the test set are considered as false negatives, as in the previous works. We confirmed that there are no duplicates in the preprocessed training set or the preprocessed test set. Table 4 shows the statistics of the DDI’13 corpus. We also count the number of instances of each class (S1 Table).

**Table 4:** The statistics of the DDIExtraction Challenge’13 corpus after preprocessing.

	Positive	Negative	Total	Ratio
Original TrainingSet	4,020	23,772	27,792	1:5.9
Zhao TrainingSet	3,840	8,989	12,829	1:2.3
Our TrainingSet	3,854	8,987	12,841	1:2.3
Original TestSet	979	4,782	5,761	1:4.9
Zhao TestSet	971	2,084	3,055	1:2.2
Our TestSet	971	2,049	3,020	1:2.1

#### Existing models for comparison

Among the existing studies that performed well on the DDI’13 corpus, the study by Kim et al. [8] used a linear kernel-based model with a rich set of lexical features. The authors proposed a two-stage method to achieve high performance. FBK-irst [9] utilized the negation scope information. A negation cue (e.g. no) is an important signal that can reverse the meaning of a particular text segment and the negation scope is the text segment that is the subject of negation. The authors of FBK-irst used an SVM classifier with a non-linear kernel.

The following neural network based models were proposed for the DDI’13 challenge. The Syntax Convolutional Neural Network (SCNN) model [10] uses word embeddings of the shortest dependency paths, position features and POS information to represent the input sentences. The Multi-Channel Convolutional Neural Network (MCCNN) model [11] uses several word embeddings for a CNN. Multiple word embeddings have more coverage than only one word embedding, because they can cover a rare word if it exists in at least one word embedding. The CNN-bioWE model [12] and the CNN-rand model [13] both implemented the Convolutional Neural Network (CNN) model combined with position embedding. The CNN-bioWE model uses word embedding trained on MEDLINE abstracts [34]. The CNN-rand model uses a random initialized word embedding matrix. The Matrix-Vector Recursive Neural Network (MV-RNN) model [17] was re-implemented for the DDI’13 Challenge. The MV-RNN model assigns a vector and a matrix to every node in a parse tree to learn the syntactic and semantic information. The Joint AB-LSTM [14] used LSTM based architectures with an attention mechanism to achieve high performance.

The SCNN model and our model report the results of both the one-stage and two-stage methods. The Joint AB-LSTM model used the one-stage method for DDI type classification and the two-stage method for detection.

#### Performance

The performance is measured using micro-averaged F1-scores. The typical F1-score is defined as F1-score = (2PR)/(P+R), where P denotes precision and R denotes recall. The micro averaged F1-score is calculated by summing the individual true positives, false positives, false negatives, and true negatives for different classes and applying the F1-score equation.

We report the performance of both our single model and our ensemble model. Our ensemble method trains the same model 10 times and sums the weight results to obtain the final result. The ensemble group’s members are structurally identical, but each ensemble member has random weight initialization. The trained ensemble members with random weight initialization produce different weight results. [35]. Since DDI extraction is a challenging task, it is difficult to reproduce the exact same result at the same stopping point (epoch) for the single model, and we mitigated this problem to some extent in the ensemble model. For the evaluation of our single model, we test its performance five times and report the average to provide more rigorous results. For the evaluation of our ensemble model, we sum the output probabilities (logits) of ensemble members, which are from the same repeated experiment. The results are provided in Table 5.

**Table 5:** Comparison between our proposed model and existing models.

	Detection			Type Classification		
	P (%)	R (%)	F (%)	P (%)	R (%)	F (%)
MV-RNN Model^*mv*^	-	-	-	52.0	48.0	50.0
CNN-rand Model^*r*^	-	-	-	69.86	56.1	62.23
Kim Model^*k*^	-	-	77.5	-	-	67.0
FBK-irst Model^*f*^	79.4	80.6	80.0	64.6	65.6	65.1
SCNN^*s*^ One-Stage Model	74.7	76.8	75.7	69.1	65.1	67.0
SCNN^*s*^ Two-Stage Model	77.5	76.9	77.2	72.5	65.1	68.6
CNN-bioWE Model^*b*^	-	-	-	75.72	64.66	69.75
MCCNN Model^*mc*^	-	-	79.0	75.9	65.2	70.2
Joint AB-LSTM Model^*j*^	86.3	75.0	80.3	73.4	69.6	71.48
Our One-Stage Model (Single)	82.1	78.5	80.1	74.4	69.3	71.7
Our One-Stage Model (Ensemble)	85.5	77.8	81.5	77.8	69.6	**73.5**
Our Two-Stage Model (Single)	80.6	84.2	81.8	77.7	66.1	71.4
Our Two-Stage Model (Ensemble)	83.6	84.0	**83.8**	79.3	67.2	72.7

Note: P, R and F denotes Precision, Recall and F1 score, respectively. Model^*mv*^ [17], Model^*r*^ [13], Model^*k*^ [8], Model^*f*^ [9], SCNN^*s*^ [10], Model^*b*^ [12], Model^*mc*^ [11], Model^*j*^ [14].

For type classification, the performance of DDI type classifier of our two-stage method is lower than that of our one-stage method. Although the performance of the detection classifier of our two-stage method is state-of-the-art, there still are false negatives in the results. The false negative instances that occur in the detection stage do not have the opportunity for classification, resulting in a slightly lower performance.

The detection classifier of our two-stage method outperforms our one-stage method in detection because the two-stage method categorizes the classes to positive and negative groups, which increases the number of training instances per class. On the other hand, the number of instances per class for the one-stage method is small because the one-stage method needs to learn five classes at a time. We also compared predicted performance of each of the four types of our model with the types of other models (S2 Table).

#### The effect of features

We performed subsequent experiments to evaluate the effectiveness of the features used in our model. We started with our best performing ensemble model, and removed the features individually while tracking any changes in performance. We test the performance of the models with different features five times and average the results. The ablation study results are shown in Table 6.

**Table 6:** Changes in our model’s performance in DDI detection by removing several features of our model.

	Removed Features	P (%)	R (%)	F (%)
Ensemble	+ All Features	83.6	84.0	**83.8**
Single	+ All Features	80.6	84.2	81.8
Single	+ All Features—Static Word Embed	69.8	81.9	75.3
Single	+ All Features—Subtree feature	78.6	84.2	81.2
Single	+ All Features—Position feature	78.0	85.5	81.4
	- Subtree feature	46.0	82.6	58.9
	- Static Word Embed	45.1	76.4	56.7

Note: P, R and F denote Precision, Recall and F1 score, respectively. We test the performance of our single model five times and average the results. For the ensemble performance, we sum the output probabilities of the ensemble members.

Without the position or subtree containment features, the F1-score slightly drops. When both features are removed, the F1-score drops sharply. To detect whether a DDI between two target drugs exists, our model needs a signal to specify the locations of the target drug pair in a sentence. The two features act as effective signals for our model. In some cases, fine-tuning the pre-trained word embeddings in the training process produced better results [36]. However, in our experimental setting, our model achieved better performance by keeping the word embedding static. This can be attributed to overfitting due to a lack of data that is required for learning. We also made a table that compares the input features of our model and the input features of other models (S3 Table).

### PK DDI data

DDIs have been recognized as an important concept in pharmacology. Wu et al. [18] released the original PK DDI corpus and Zhang et al. [33] later used the same data format as the DDI’11 corpus. We utilized the second version of the PK DDI data, which is more refined than the original (https://sbmi.uth.edu/ccb/resources/ddi.htm). The PK DDI corpus was manually created from 428 Medline abstracts. The authors of the PK DDI corpus differentiated the data into *in vivo* DDIs and *in vitro* DDIs. The *in vivo* PK DDI studies focus on cases where metabolism and efficacy of a drug are influenced by another drug. The *in vitro* DDI studies usually determine whether a target drug is an inhibitor or an inducer of other drugs. Because all the data is limited in size, we did not perform negative instance filtering. Table 7 displays the statistics of the PK DDI corpus data.

**Table 7:** The statistics from the PK DDI corpus after preprocessing.

	Positive	Negative	Total	Ratio
in vivo DDI training data	781	2,809	3,590	1:3.5
in vivo DDI test data	213	676	889	1:3.1
in vitro DDI training data	544	3,984	4,528	1:7.3
in vitro DDI test data	146	837	1,015	1:5.7

#### Existing models for comparison

Zhang et al. [33] used a refined-semantic class annotation method which replaces several important terms related to the PK DDI process with more generic terms. Zhang et al. implemented the all-paths graph kernel method which uses dependency graphs that represent sentence structures [37]. In addition to the semantic class annotation, Zhang et al. also used predicate-argument structures (PASs) in place of the dependency parser result. We denote the dependency parsing version results as DEP_ReSC and the PAS version results as PAS_ReSC, both of which results are obtained from the previous study [33].

The PK DDI corpus has only baseline results tested by the authors of the data. We tried to use the baseline results of the DDI’13 corpus for the PK DDI corpus. However, the existing studies that released the code provide the pre-processing code part only for the DDI’13 corpus or lack details on how to pre-process data other than the DDI’13 corpus. Also, machine learning models that do not go through hyper-parameter adjustments will obtain lower performance; therefore, we report only the baseline results obtained from the previous study [33].

#### Performance

The performance is measured using F1-score. We report the performance of both our single and ensemble models. The performance of our single model is measured based on the average results of five repeated experiments. For the ensemble performance, we sum the output probabilities of the 10 ensemble members. The performance results are listed in Tables 8 and 9.

**Table 8:** Comparison of *in vivo* PK DDI results of our model and those of existing models.

	Precision (%)	Recall (%)	F1-score (%)
PAS_ReSC [31]	84.8	68.5	75.8
DEP_ReSC [31]	80.8	83.1	81.9
Our Model (Single)	82.1	83.3	82.6
Our Model (Ensemble)	85.0	82.6	**83.8**

**Table 9:** Comparison of *in vitro* PK DDI results of our model and those existing models.

	Precision (%)	Recall (%)	F1-score (%)
PAS_ReSC [31]	74.8	62.5	68.1
DEP_ReSC [31]	70.7	67.9	69.3
Our Model (Single)	80.3	65.9	72.3
Our Model (Ensemble)	81.2	67.9	**74.0**

Our model outperforms other baseline models on both the *in vitro*, and *in vivo* datasets. Both datasets were difficult to use for training because their size is smaller than that of the DDI’13 corpus. While the model of Zhang et al. [33] improves performance using re-annotated data, our model achieves better performance without using re-annotated data.

#### The effect of features

We remove features individually to evaluate the effectiveness of the features and report changes in performance. We test the performance of our model with different features five times and report the average of the results. The ablation study results are in Table 10. As in the case of the DDI’13 corpus, the more features are removed, the lower the overall score.

**Table 10:** Performance changes of our model on the PK DDI in vivo dataset by removing features.

	Removed Features	P (%)	R (%)	F (%)
Ensemble	+ All Features	85.0	82.6	**83.8**
Single	+ All Features	82.1	83.3	82.6
Single	+ All Features—Subtree feature	78.8	84.2	81.3
	- Position feature	55.7	74.4	62.8
	- Static Word Embed	51.8	70.7	59.5

Note: P, R and F denotes Precision, Recall and F1 score, respectively. The same experiment was repeated five times and the results were averaged.

## Discussion

### Robustness of our model

The preprocessing method contributes to performance improvement by filtering numerous noisy instances. We examined the performance of our two-stage detection classifier (single) on the original DDI’13 Challenge data to determine the impact of preprocessing methods on performance and the robustness of our model. Our model trained on the non-preprocessed data obtains an F1-score of 80.3%; however, our model trained on the preprocessed data achieved an F1-score of 81.8%. Our model trained on the non-preprocessed data suffered a 1.5% drop in performance. Although our model does not depend heavily on preprocessing, a very simple preprocessing method such as the one applied to our data, may improve the performance.

### Regularization analysis

The original tree-LSTM model [27] used l2 regularization, but we implemented recurrent dropout [29] to achieve better performance. To compare l2 regularization and recurrent dropout, we searched the best λ value for l2 regularization. We report the performance of the two-stage detection classifier (single) using the l2 regularization technique. The best λ value for l2 regularization is 1.0 and the F1-score of the two-stage detection classifier (single) using l2 regularization model is 79.8%, while that of our recurrent dropout model is 81.8%.

### Error analysis

We examine the cases where our best ensemble based model fails to detect an interaction between target drugs. We explain the three most common error cases below and miscellaneous errors in the “ETC” group. We provide examples of the three most common cases in Table 11.

**Table 11:** Examples of three common types of error cases.

Num	DDI	Sentence
1	True	There is usually complete cross-resistance between PURINETHOL_*drug*0_ (mercaptopurine_*drug*1_) and TABLOID_*drug*2_ brand Thioguanine_*drug*3_.
2	True	The bioavailability of SKELID_*drug*0_ is decreased 80% by calcium_*drug*1_, when calcium_*drug*2_ and SKELID_*drug*3_ are administered at the same time, and 60% by some aluminum_*drug*4_—or magnesium_*drug*5_ -containing antacids_*drug*6_, when administered 1 hour before SKELID_*drug*7_.
3	False	The drug interaction between proton pump inhibitors_*drug*0_ and clopidogrel_*drug*1_ has been the subject of much study in recent years.

Note: Underlined drug names are target drugs.

Case 1. *When a strong hint for a positive instance does not exist in the training set*.

It is difficult to accurately detect interaction if there is an expression that was not present in the training process. In such a case, a detection classifier is set only to find DDIs based on the words contained in the word embedding. However, it is difficult to find DDIs because the word embedding is trained on unsupervised language models. For example, the instance number 1 in Table 11 has the strong positive indicator “cross-resistance”, but the word does not appear in the training set. Errors of the first case constitutes 5% of all the errors.

Case 2. *When a sentence has a complex structure and the target drugs are positioned far from the primary information*.

A complex sentence structure consists of at least one independent and possibly many subordinate clauses. In this case, the words around the drug pairs are insufficient to accurately detect the interaction since the subordinate clauses do not contain important information. For example, the instance number 2 in Table 11 has a complex sentence structure. The sentence is semantically divided at the “,and 60%” part, because the clause immediately before the “,and 60%” part is a subordinate clause and the “, and 60%” part is associated with the first independent clause. The NLP parser can divide clauses, but it does not give information about which clause contains the main information (e.g., “SKELID is decreased” by using the target drug). Errors of the second case account for 17% of the total errors. The errors in the first and second cases are false negatives.

Case 3. *Relations are described using unclear terms and falsely recognized as positive instances*.

There are several sentences which have structures or expressions that are similar to true instances but are actually false instances. For example, instance number 3 in Table 11 states that the interaction between the two drugs has been studied in previous researchers but the existence of the interaction is not described conclusively in the sentence. Our model misclassifies the instance because the strong relation word “interaction” appears in the sentence. Errors of the third case composes 46% of the total errors. The third case has only false positives.

Last, the “ETC” case is a set of error instances that do not belong to the three cases mentioned above. The instances of the “ETC” case do not share apparent similarities. The “ETC” case comprises 32% of the total errors.

There is a are total of four error cases, but the solutions for each error cases are almost the same. Using a larger amount of data or applying the attention mechanism can prevent our model from misclassifying interactions. Large data will reduce the variance of our model, and possibly reduce the number of error cases mentioned above. Currently we use the DDI challenge’13 corpus, but we expect to improve performance when we apply our model to larger data in the future. The attention mechanism helps a neural network model to locate the important part of the sentence in the training process [38]. However, applying the attention mechanism to the recursive neural network models is a difficult task and we leave it as a future work.

## Conclusion

Our recursive neural network model achieved better performance than the existing models on both the DDI’13 corpus and PK DDI corpus. We implemented the tree-LSTM architecture to understand the natural language sentences. We showed that a position feature and a subtree containment feature can effectively locate the target drugs in a sentence. Our recursive neural network model outperformed the state-of-the-art model by 4.4% and 2.8% in the detection and classification tasks, respectively. We also tested our recursive neural network model on *in vivo* and *in vitro* DDI data separately. Our detection model performed 2.3% and 6.7% better on *in vivo* and *in vitro* data, respectively. As the volume of published information rapidly grows, techniques for accurately extracting information from the literature become increasingly more important. We hope that our model can be a useful part of the solution to handling overwhelming amounts of data. The source code of our detection model is available at https://github.com/arwhirang/DDI-recursive-NN.

## Supporting information

S1 Table The number of instances in each of the four types of the DDIExtraction Challenge’13 corpus after preprocessing. (DOCX)

S2 Table The F1-score comparison result of the individual classes. (DOCX)

S3 Table The comparison of the input features used in our method with those of other baselines. (DOCX)
