# Supplementary material for: Drug drug interaction extraction from the literature using a recursive neural network
Source: PLoS One. 2018 Jan 26;13(1):e0190926. doi: 10.1371/journal.pone.0190926 (PMC5786304; doi:10.1371/journal.pone.0190926)
Supplement: S1 Table — (DOCX) [file pone.0190926.s001.docx]

**Supplementary Table 1.** The number of instances in each of the four types of the DDIExtraction Challenge’13 corpus after preprocessing

|  | *Original Training Set* | *Original Test Set* | *Our Training Set* | *Our Test Set* |
| --- | --- | --- | --- | --- |
| *Mechanism* | *1,319* | *302* | *1,260* | *301* |
| *Effect* | *1,687* | *360* | *1,592* | *357* |
| *Advice* | *826* | *221* | *814* | *221* |
| *Int* | *188* | *96* | *188* | *92* |
| *Positive* | *4,020* | *979* | *3,854* | *971* |
