# Supplementary material for: Drug drug interaction extraction from the literature using a recursive neural network
Source: PLoS One. 2018 Jan 26;13(1):e0190926. doi: 10.1371/journal.pone.0190926 (PMC5786304; doi:10.1371/journal.pone.0190926)
Supplement: S2 Table — (DOCX) [file pone.0190926.s002.docx]

**Supplementary Table 2.** The F1-score comparison result of the individual classes.

|  | *Mechanism (%)* | *Effect (%)* | *Advice (%)* | *Int (%)* |
| --- | --- | --- | --- | --- |
| *FBK-irst Model* | *67.9* | *62.8* | *69.2* | ***54.7*** |
| *Kim Model* | *69.3* | *66.2* | *72.5* | *48.3* |
| *MCCNN Model* | *72.2* | *68.2* | *78.0* | *51.0* |
| *Our One-Stage Model (single)* | *73.7* | *68.5* | *76.6* | *43.9* |
| *Our One-Stage Model (Ensemble)* | ***75.1*** | ***72.9*** | ***82.7*** | *43.5* |

Note: We use the one-stage model only because our one-stage model’s individual class performance is better than that of the two-stage model.
