# Supplementary material for: Drug drug interaction extraction from the literature using a recursive neural network
Source: PLoS One. 2018 Jan 26;13(1):e0190926. doi: 10.1371/journal.pone.0190926 (PMC5786304; doi:10.1371/journal.pone.0190926)
Supplement: S3 Table — (DOCX) [file pone.0190926.s003.docx]

**Supplementary Table 3.** The comparison of the input features used in our method with those of other baselines.

| Used Input Features | Kim model^k^ | SCNN model^s^ | MV-RNN model^mv^ | Our model |
| --- | --- | --- | --- | --- |
| Word | ✓ (U, B, T) * | ✓ | ✓ | ✓ |
| Parse tree | ✓ | - | ✓ | ✓ |
| Position | ✓ | ✓ | - | ✓ |
| Subtree containment | - | - | - | ✓ |
| Word embedding | - | ✓ (Syntactic) * | ✓ | ✓ |
| Dependency graph | ✓ (U, B, T) * | ✓ | - | - |
| Word pair | ✓ | - | - | - |
| NPC * | ✓ | - | - | - |
| Part of speech | ✓ | ✓ | - | - |
| Biomedical semantic type (MetaMap) | - | ✓ | - | - |

Note: ✓ (check) means that the feature is used, while - (hyphen) means that the feature is not used. NPC * denotes the “Noun Phrase Coordination” feature used in the Kim model^k^. (U, B, T) * denotes the unigram, bigram and trigram. (Syntactic) * denotes the syntactic word embedding used in the SCNN model^s^. The FBK-irst model^f^ used more than 10 different contextual and shallow linguistic features. We do not list the features of the FBK-irst model, since the list of features may confuse the readers. Other deep learning based models use only word features and word embedding features.
